# Functional status and depressive symptoms in relation to bone mineral density and osteoporosis in hospitalized older adults: a cross-sectional study

**DOI:** 10.1186/s12877-026-08424-4

**Published:** 2026-10-06

**Authors:** Stylianos Kopanos, Sandra Scheel, Bettina Eggert, Alexander Rübberdt, Ulrich Thiem, Joachim Feldkamp

**Affiliations:** 1 Academic Department of Endocrinology, Diabetes and Infectiology, Klinikum Bielefeld, Medical School, University Medical Centre East Westphalia-Lippe Bielefeld University, Bielefeld, Germany; 2 Department of Orthopaedics and Trauma Surgery, University Medical Center East Westphalia-Lippe, University of Bielefeld, Bielefeld, Germany; 3 Department of Geriatrics, Medical School OWL, University of Bielefeld, Bielefeld, Germany

**Keywords:** Osteoporosis, Bone mineral density, Geriatric assessment, Depression, Functional status

## Abstract

**Introduction:**

Osteoporosis is a major cause of morbidity and fractures among older adults, contributing substantially to disability, hospitalization, and mortality. This cross-sectional study investigated the associations of depressive symptoms and functional status with bone mineral density (BMD) and osteoporosis in hospitalized older adults.

**Methods:**

We analyzed 1,048 hospitalized patients aged ≥ 70 years who underwent dual-energy X-ray absorptiometry (DXA). Depressive symptoms (GDS-15) and functional status (Barthel Index) were extracted from routine geriatric assessments. Group comparisons, multivariable linear regression, and logistic regression analyses were performed after adjustment for demographic, clinical, and endocrine covariates.

**Results:**

Lower Barthel Index scores and higher depressive symptom burden were independently associated with lower hip BMD, whereas associations with lumbar spine BMD were less consistent. Although statistically significant, the observed effect sizes were modest. The multivariable model explained a moderate proportion of BMD variability (adjusted R² = 0.22).

**Conclusion:**

Functional and psychological characteristics were independently associated with bone mineral density in hospitalized older adults. Given the cross-sectional design, causal relationships cannot be established. These findings support the potential value of incorporating comprehensive geriatric assessment into osteoporosis evaluation to improve identification of older adults at increased fracture risk.

## Introduction

Osteoporosis is a widespread and debilitating condition among the elderly, characterized by reduced bone mineral density (BMD) and an increased susceptibility to fractures. These fractures often lead to a cascade of adverse outcomes, including decreased mobility, loss of independence, prolonged hospitalization, and higher mortality rates [1, 2]. While traditional risk factors such as age, female gender, vitamin D deficiency, and hormonal imbalances are well-documented in osteoporosis research, geriatric-specific parameters remain underexplored despite their potential influence on bone health [3, 4].

Emerging evidence suggests that cognitive function, depression, functional status, and hospitalization duration may contribute significantly to osteoporosis risk. Cognitive decline, assessed through tools such as the Mini-Mental State Examination (MMSE), has been associated with reduced physical activity, poor nutritional intake, and an increased risk of falls, all of which may contribute to bone loss [5, 6]. Similarly, depression, as measured by the Geriatric Depression Scale (GDS-15), has been linked to lower physical activity levels, decreased sun exposure, and altered calcium homeostasis, which may exacerbate osteoporosis progression [7]. The interplay between mental health and bone health is often overlooked, yet depression and cognitive impairment can significantly affect adherence to osteoporosis prevention strategies, including diet, supplementation, and physical activity [8, 9].

Functional status and independence in activities of daily living (ADL), commonly assessed using the Barthel Index, play a critical role in maintaining skeletal integrity [10]. Reduced functional independence is associated with reduced weight-bearing activity, sarcopenia, and an increased likelihood of falls, all of which contribute to bone loss and fracture risk [11]. Hospitalization, particularly prolonged or recurrent admissions, is another underrecognized factor that may impact osteoporosis risk [12, 13]. Extended bed rest and immobilization during hospitalization accelerate bone resorption, particularly in older adults, leading to rapid BMD decline. Patients experiencing long-term hospital stays or recurrent admissions may also have underlying frailty, comorbid conditions, and poor nutritional intake, further compounding their osteoporosis risk [14]. Given the aging population and the increasing prevalence of osteoporosis, it is crucial to examine to what extent these geriatric and hospitalization factors influence BMD and fracture risk [15]. These geriatric domains frequently coexist and may reinforce one another through shared biological, functional, and behavioral pathways. Their combined evaluation may therefore provide a more comprehensive understanding of bone health than considering each factor separately.

Despite the well-established biological determinants of osteoporosis, the contribution of geriatric functional and psychological factors remains insufficiently characterized. Previous studies have primarily examined cognitive impairment, depressive symptoms, or functional decline separately, and their findings have been inconsistent across different older populations. Moreover, relatively few investigations have evaluated these domains simultaneously in hospitalized older adults, despite their frequent coexistence and potential interactions. Consequently, the independent and combined associations of depressive symptoms and functional status with bone mineral density remain incompletely understood. Addressing these knowledge gaps may facilitate a more comprehensive approach to osteoporosis assessment and management in geriatric patients.

Given these knowledge gaps, a comprehensive evaluation of multiple geriatric domains within the same hospitalized population may improve our understanding of factors associated with bone health. The primary aim of this study was to investigate the associations between depressive symptoms, functional status, and bone mineral density in hospitalized older adults. Furthermore, multivariable analyses were performed to identify independent geriatric factors associated with reduced bone mineral density after adjustment for established clinical and endocrine risk factors. The primary outcome was bone mineral density measured by dual-energy X-ray absorptiometry (DXA) at the hip and lumbar spine.

## Methods

### Study design and population

This retrospective cross-sectional study was designed to investigate the association between functional domains as assessed by geriatric assessment and osteoporosis risk in elderly inpatients who underwent bone mineral density (BMD) measurements. Data were systematically retrieved from hospital electronic medical records, ensuring a comprehensive dataset that included clinical, functional, and demographic variables. The electronic records system was queried for 1,155 patients aged 70 years or older who underwent DXA-based BMD assessments at the hip and lumbar spine. Extracted information included demographic data such as age, sex, and body mass index (BMI), as well as clinical history involving osteoporosis diagnoses, cognitive impairment, depression, and functional impairment. Additionally, functional assessments, including the Geriatric Depression Scale (GDS-15) for depression severity, and the Barthel Index for functional status, were recorded. Hospitalization details, including length of hospital stay and history of previous admissions, were also collected and evaluated.

All geriatric assessments (GDS-15 and Barthel Index) and DXA examinations were performed during the same hospitalization as part of routine comprehensive geriatric assessment. Because data were collected retrospectively from a single hospitalization, temporal relationships between functional status and bone mineral density could not be established.

### Inclusion and exclusion criteria

The study population consisted of elderly patients hospitalized for different reasons in our geriatric clinic or, if fractures were present, in our department for geriatric trauma surgery. All patients were treated from geriatricians. Inclusion criteria were age > 70 years DXA-based BMD evaluation and documented cognitive, depression, and functional status assessments. Patients were excluded if they had incomplete or missing BMD measurements or lacked data on cognitive, depression, or functional status. Additional exclusion criteria included the presence of advanced chronic conditions known to significantly affect bone metabolism, such as severe chronic kidney disease (eGFR < 30 mL/min/1.73 m²), active malignancies with bone metastases, or chronic inflammatory diseases like rheumatoid arthritis and systemic lupus erythematosus. Patients on long-term corticosteroid therapy exceeding 5 mg of prednisolone equivalent for more than six months or those already receiving osteoporosis treatment at baseline, such as bisphosphonates, denosumab, or parathyroid hormone analogues, were also excluded. Furthermore, individuals with severe neurological impairments affecting mobility, such as advanced Parkinson’s disease or recent stroke with significant disability, were not included. These criteria were selected to minimize major confounding on bone metabolism but may limit generalizability.

A total of 1,155 patients were initially identified from the osteoporosis registry.

**Fig. 1:**
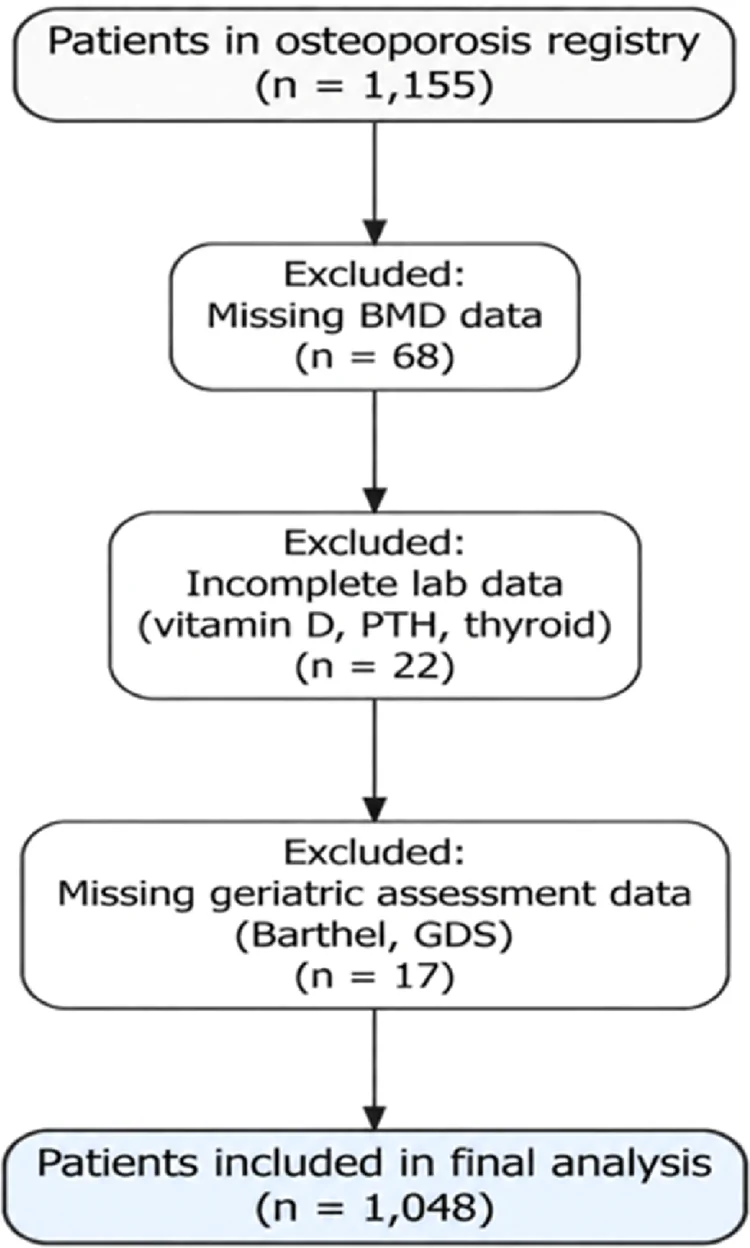
Participant selection flow diagram

After data screening, 68 patients were excluded due to missing BMD measurements, 22 due to incomplete laboratory parameters (vitamin D, PTH, or thyroid hormones), and 17 due to missing geriatric assessment data (Barthel Index or GDS-15). The final analytical sample therefore comprised 1,048 patients with complete datasets for all main variables. The detailed inclusion and exclusion process is presented in participant selection flow diagram (Fig. 1).

Following application of the inclusion and exclusion criteria, a final cross-sectional study of elderly hospitalized patients was selected for analysis. Comparative analyses were performed across subgroups defined by cognitive performance, depression severity, and functional status to explore their associations with BMD and osteoporosis risk in the overall population.

### Ethical approval

For this study was obtained from the Ethics Committee of Westphalia-Lippe, Germany (2025-443-f-S), and all participants provided written informed consent prior to enrollment.

### Bone mineral density measurement

Bone mineral density was measured using dual-energy X-ray absorptiometry (DXA, Lunar™, GE HealthCare) at two skeletal sites: Hip BMD, which primarily reflects cortical bone health and Lumbar spine BMD, which is predominantly composed of trabecular bone and may be more sensitive to metabolic and functional changes.

Osteoporosis was defined according to the World Health Organization (WHO) classification, with a T-score ≤ -2.5 SD indicating osteoporotic bone loss. Osteopenia was categorized as a T-score between − 1.0 and − 2.5 SD, while normal bone density was defined as a T-score above − 1.0 SD. DXA scans were conducted following standardized protocols, with regular calibration of equipment to ensure measurement accuracy.

As the bone mineral density (BMD) was treated as a continuous dependent variable, all analyses were performed in the direction of “factors associated with BMD.” This analytical approach was chosen to preserve the continuous information of the bone density values and to capture subtle linear associations. Alternatively, BMD could have been categorized (normal, osteopenia, osteoporosis) and analyzed in relation to geriatric parameters, but this would have entailed a potential loss of statistical power and interpretability.

Hip and lumbar BMD are reported as T-scores from DXA. Z-scores were not available in the exported dataset and could not be derived without instrument-specific age- and sex-matched reference data. No analyses in g/cm² were performed.

### Geriatric assessment

To evaluate the impact of cognitive function, depression, and functional status on osteoporosis risk, the following geriatric assessments were conducted:

Depression (Geriatric Depression Scale, GDS): The Geriatric Depression Scale (GDS) was used to assess depression severity in the elderly population. The GDS is a self-reported questionnaire, with scores categorized as follows: No Depression (GDS 0–3), Mild to Moderate Depression (GDS 4–6), Significant Depression (GDS ≥ 7).

Functional Status and Independence (Barthel Index): Functional status and independence in activities of daily living (ADL) were assessed using the Barthel Index. Scores range from 0 to 100, with higher scores reflecting greater independence. Patients were grouped based on their functional status as follows: Independent / minimally dependent (Barthel Index > 60) – Independent or requiring minimal assistance, Moderate dependency (Barthel Index 30–60) – Partial dependence on caregivers, Severe dependency (Barthel Index < 30) – Highly dependent on assistance for daily activities.

These classifications were used to examine whether prolonged hospital stays were associated with greater reductions in BMD due to reduced functional capacity, higher frailty, or other underlying medical conditions.

### Statistical analysis

A comprehensive statistical approach was used to determine the relationship between geriatric parameters and BMD outcomes: Descriptive statistics summarized baseline characteristics, including age, sex, GDS levels and Barthel Index scores. Group comparisons were performed using one-way ANOVA for normally distributed variables to compare BMD levels among different cognitive, functional status, and depression groups and Kruskal-Wallis or Mann-Whitney U tests where assumptions of normality were not met. Normality was assessed using QQ-plots and Shapiro–Wilk tests. Non-parametric tests were used where distributions violated normality.

Linear regression models were used to assess the independent impact of cognitive decline, depression severity, and functional limitations on BMD, adjusting for age, sex, BMI, and hospitalization duration.

Categorical predictors from the geriatric assessment (e.g., depressive mood [yes/no], cognitive impairment [present/absent], functional dependency [yes/no]) were included in the model as dummy variables, with the non-impaired or normal category serving as the reference.

Before applying the models, scatterplots and residual analyses were used to confirm approximate linearity and homoscedasticity assumptions. Regression coefficients (β), 95% confidence intervals (CI), and p-values were reported for each predictor. Model fit was assessed by the adjusted R².

Logistic regression analyses were performed to assess the association between clinical and functional parameters and the presence of osteoporosis (defined as T-score ≤ − 2.5).

For each categorical variable (e.g., depression severity, cognitive impairment, functional status, living situation), the reference category was defined as the clinically normal or most favorable condition (e.g., non-depressive mood, normal cognition, independent functional status, living at home), corresponding to an odds ratio (OR) of 1.0.

Both crude (unadjusted) and adjusted models were calculated. The adjusted models included age, sex, BMI, serum vitamin D, and PTH as covariates. This approach allowed the estimation of independent associations between each assessment parameter and osteoporosis risk. Results are reported as odds ratios (OR) with corresponding 95% confidence intervals (CI) and p-values. Model fit was evaluated using the Nagelkerke R² and the Hosmer–Lemeshow goodness-of-fit test.

Missing data were handled using complete-case analysis. Only participants with available data for all variables included in a given statistical model were analyzed. No imputation procedures were performed.

Interaction terms between key variables were tested to explore potential effect modification. Specifically, interactions between age and sex, depression severity and functional status, and cognitive performance and vitamin D levels were examined. A significance threshold of *p* < 0.10 was adopted for interaction testing because interaction effects generally have lower statistical power than main effects, particularly in observational studies. This threshold has been recommended to reduce the likelihood of overlooking potentially meaningful effect modification. All primary hypothesis testing remained based on the conventional two-sided significance level of *p* < 0.05.

All statistical analyses were conducted using RStudio (version 2024.12.0 + 467, Boston, MA, USA).

## Results

The retrospective cross-sectional study consisted of 1,048 patients, with a mean age of 85.0 ± 7.1 years; range 72–96 years (80.1% female, 19.9% male). 7% were operated due to fractures, while 93% were treated conservatively. Before inpatient admission, 65% of the patients studied stayed at their own domicile, while 35% lived in a nursing home.

**Fig. 2:**
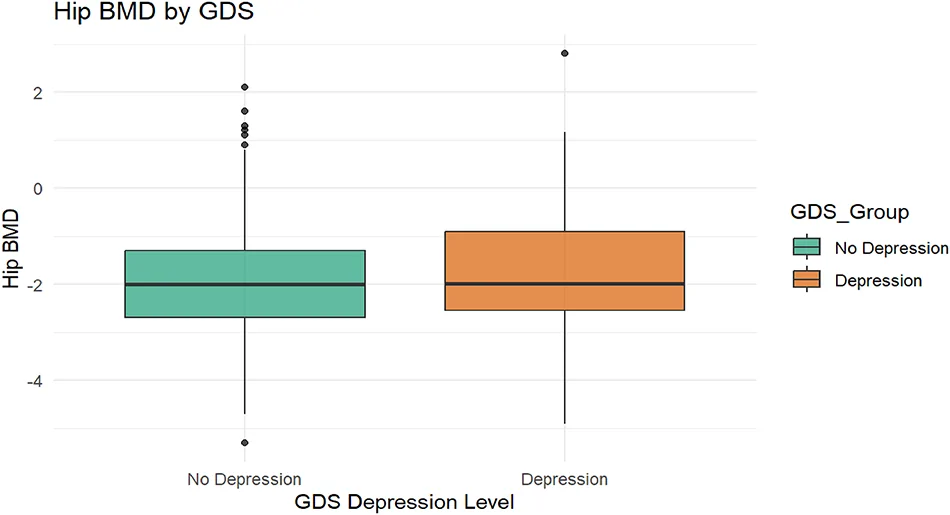
Depression Scaling vs. Hip BMD. Boxplots illustrate differences in hip bone mineral density (BMD) between patients with and without depressive symptoms according to the Geriatric Depression Scale (GDS). Patients classified as “depressed” showed lower median hip BMD compared with non-depressed individuals, indicating a group difference rather than a continuous correlation

The associations between geriatric assessment parameters for depression severity, cognitive function, functional status and bone mineral density were evaluated using group comparisons, correlation analyses, and multivariable regression models. Based on the Geriatric Depression Scale (GDS-15; cut-off ≥ 6 for depressive symptoms), higher GDS-15 scores were associated with lower hip BMD (mean difference − 0.05 g/cm², 95% CI − 0.19 to − 0.01; *p* = 0.027). No significant association was observed between depressive symptoms and lumbar spine BMD (Spearman’s ρ = −0.02, *p* = 0.565), and hip T-scores did not differ significantly between depression categories (Mann–Whitney U = 584.5, *p* = 0.94) (Fig. 2).

**Fig. 3:**
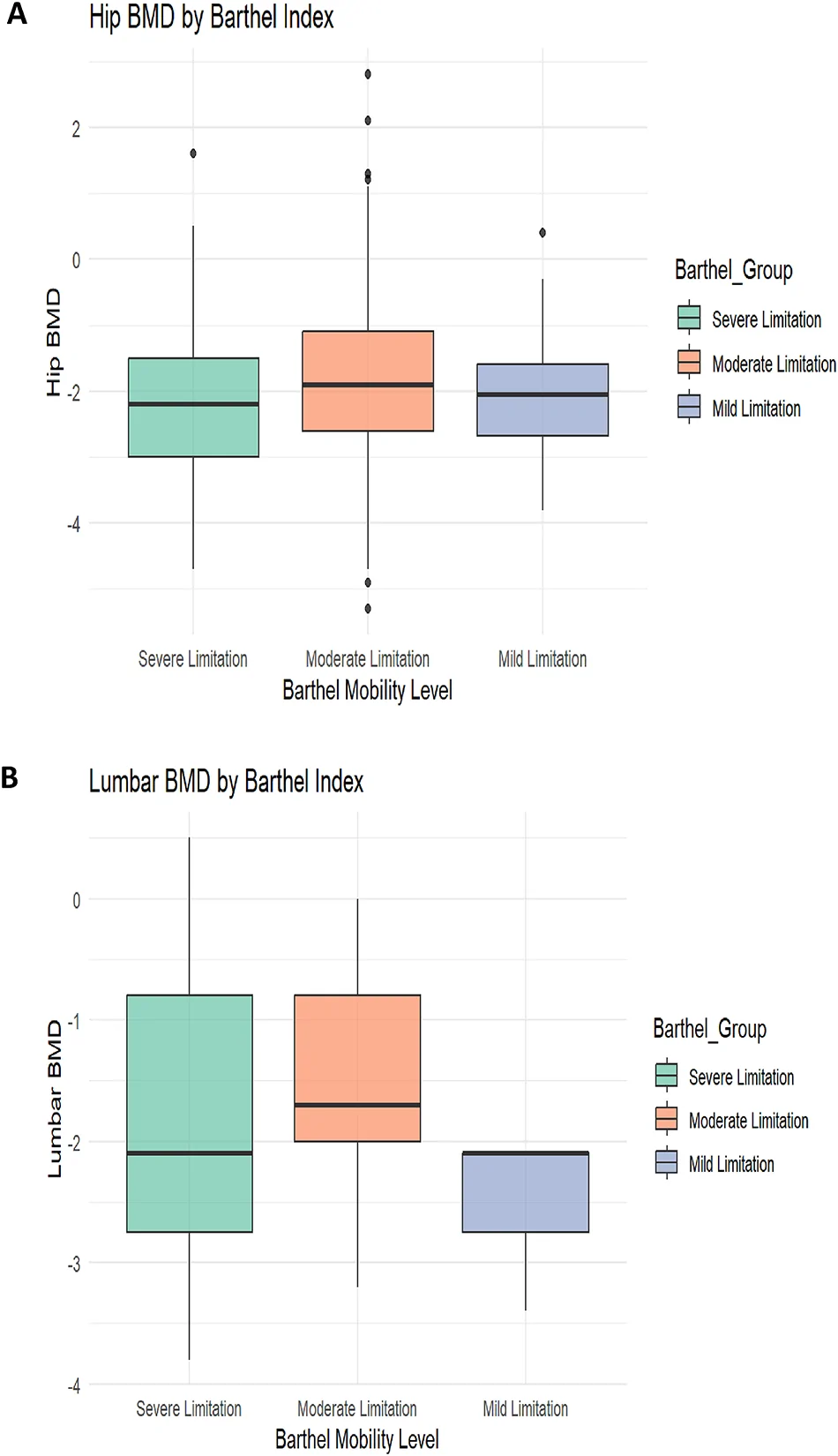
**A** Hip BMD by Barthel Index. This boxplot highlights the relationship between functional status (Barthel Index) and hip BMD. Patients with severe functional dependency have significantly lower BMD compared to those with moderate or mild limitations. Patients with better functional status had higher hip BMD. **B** Lumbar BMD by Barthel Index. A similar trend is observed for lumbar BMD, where patients with severe functional dependence have the lowest values. The median lumbar BMD appears highest in the moderate limitation group, suggesting that moderate functional status may offer some protective effect compared to severe restriction. However, the mild limitation group shows a slightly lower BMD than the moderate group, which might indicate that other factors, such as comorbidities, could influence lumbar bone density

Participants with more severe cognitive impairment had progressively lower hip T-scores (normal cognition: −1.85 ± 1.20; mild impairment: −2.09 ± 1.15; severe impairment: −2.27 ± 1.26). Group differences were statistically significant (Kruskal–Wallis H = 6.94, *p* = 0.031; Fig. 3), whereas lumbar spine BMD did not differ significantly across cognitive categories (H = 2.40, *p* = 0.30).

Higher Barthel Index scores were weakly associated with higher hip BMD (Spearman’s ρ = 0.14, *p* = 0.001; Fig. 3). No significant association was observed between the Barthel Index and lumbar spine BMD (ρ = 0.05, *p* = 0.25). Patients with severe functional dependency had significantly lower lumbar and hip BMD than those with mild or moderate dependency (95% CI − 2.18 to − 1.67, *p* < 0.001).

Patients aged 80–90 years exhibited significantly lower hip BMD than those younger than 80 years (estimate = -1.02, 95% CI: -1.90 to -0.14, *p* = 0.024), whereas patients over 90 years of age showed a similar trend but without statistical significance (*p* = 0.128).

Statistical models further explored the interplay of geriatric parameters and osteoporosis risk. In the analysis population, both operated and non-operated patients were included. Compared with non-operated individuals, those who had undergone an operation (post-fracture) showed higher odds of exhibiting mild cognitive impairment (OR = 4.50, 95% CI: 1.17–17.33). This finding indicates an association between surgical status and cognitive function within the population of the study, without implying causality. Patients with normal cognition had a 2.36 times higher likelihood of requiring a post-fracture operation (OR = 2.36, 95% CI: 1.08–5.13). Analysis of variance (ANOVA) confirmed that functional status had a statistically significant effect on BMD (95% CI: 27.21–34.94, *p* < 0.01).

**Fig. 4:**
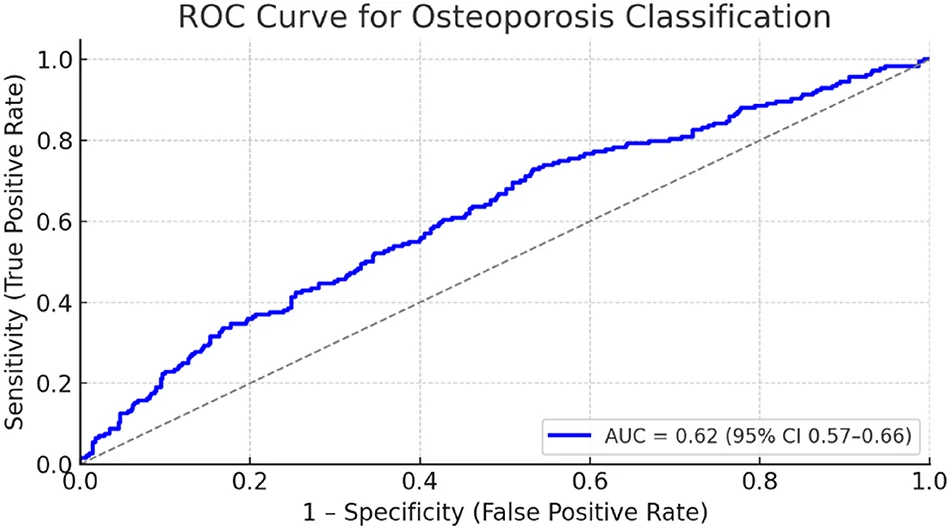
ROC Curve for osteoporosis classification. T The curve plots sensitivity (true positive rate) against 1 – specificity (false positive rate). Outcome was defined as osteoporosis (T-score ≤ − 2.5 at hip or lumbar spine). The multivariable logistic model (age, sex, Barthel Index, GDS, vitamin D, PTH) yielded AUC = 0.62 (95% CI: 0.57–0.66); the diagonal gray line denotes a random classifier (AUC = 0.5)

Logistic regression analysis for osteoporosis classification yielded an AUC of 0.62 (95% CI 0.58–0.69), indicating modest discriminative ability. The adjusted model included age, sex, Barthel Index, GDS-15, serum vitamin D, and PTH (Fig. 4).

In multivariable linear regression analyses, age, depressive symptoms, and Barthel Index showed the strongest independent associations with hip BMD. Serum vitamin D was not significantly associated with hip or lumbar spine BMD.

Additionally, fallers had significantly lower Barthel Index scores (mean: 48.3) compared to non-fallers (mean: 67.1) (**Supp.** Figure 1).

Overall, lower functional status, greater depressive symptom burden, and poorer cognitive performance were associated with lower hip BMD, whereas the observed effect sizes were modest and lumbar spine associations were less consistent.

## Discussion

This study underscores the significant impact of geriatric function as displayed by geriatric assessment on osteoporosis risk, highlighting the interplay between depression and functional status, in determining bone health outcomes [16]. Our findings demonstrate that depression, as measured by the Geriatric Depression Scale (GDS-15), is significantly associated with lower hip bone mineral density (BMD). This supports prior research indicating that depression has been associated with alterations in bone metabolism, likely through increased levels of inflammatory cytokines and reduced engagement in weight-bearing activities [17]. These findings suggest that depressive symptoms may represent an important clinical marker associated with osteoporosis risk [18]. While several studies have examined bone mineral density (BMD) in community-dwelling older adults, few have evaluated the combined impact of functional, cognitive, and emotional status in hospitalized patients — a population at particularly high fracture risk [19]. Our findings therefore provide novel insight into the geriatric functional and psychological determinants of bone health within geriatric care settings.

Functional status, assessed using the Barthel Index, exhibited a positive association with BMD. Patients with severe functional dependency had significantly lower lumbar and hip BMD than those with moderate or mild limitations [20, 21]. This finding aligns with evidence that physical activity supports bone remodeling and preserves skeletal integrity [22]. Participants with a history of falls also exhibited significantly lower Barthel Index scores, suggesting that impaired functional independence and mobility are closely linked to fall occurrence and may contribute to increased fracture vulnerability [23]. Although causality cannot be inferred, these findings emphasize the importance of preserving functional capacity in older adults [24].

Bone health in older adults is multifactorial and is influenced by numerous biological, clinical, behavioral, and environmental determinants beyond those evaluated in the present study [25–27]. The observed association between depressive symptoms and reduced bone mineral density may reflect both behavioral and biological mechanisms. Behavioral pathways include lower physical activity and poorer nutritional intake commonly observed in individuals with depressive symptoms. Biological mechanisms may involve dysregulation of the hypothalamic-pituitary-adrenal axis resulting in increased cortisol secretion, together with elevated inflammatory mediators such as interleukin-6 (IL-6), both of which have been associated with impaired bone remodeling in previous studies. These alterations may contribute to decreased bone formation and increased bone resorption, leading to lower bone mineral density in depressed individuals. However, because of the cross-sectional design, these mechanisms remain speculative and were not directly evaluated in the present study. The overall multivariable model demonstrated only moderate explanatory and discriminative ability (adjusted R² = 0.22; AUC = 0.62), indicating that although geriatric functional parameters contribute independently to bone health, a substantial proportion of BMD variability remains attributable to additional biological, lifestyle, and clinical determinants not captured in the present analyses [28].

These findings have several important clinical implications. Routine osteoporosis screening should integrate comprehensive geriatric assessments, including depression (GDS-15) and functional status (Barthel Index), to better identify high-risk individuals [27, 29]. Early mobility interventions for hospitalized elderly patients could mitigate bone loss by encouraging weight-bearing activities [30]. Moreover, proactive management of depression and cognitive decline should be considered as part of comprehensive geriatric care, given their indirect yet meaningful contributions to fracture risk [31–33]. From a clinical standpoint, integrating cognitive and emotional assessments into osteoporosis screening protocols could facilitate early identification of frail patients at risk for accelerated bone loss. Rehabilitation strategies and psychosocial interventions may thus warrant further investigation [34].

A major strength of this study is its high number of patients, comprehensive assessment of multiple geriatric parameters in relation to BMD, integrating physical and psychological factors to provide a multidimensional perspective on osteoporosis risk. The use of validated clinical scales (Barthel Index, GDS-15) enhances the reliability of our findings. In our retrospective cross-sectional study, the multivariable logistic model showed only moderate discrimination for osteoporosis, underscoring the multifactorial nature of bone loss in older adults. Predictive performance remained modest, indicating that functional and psychological variables alone cannot explain most of the variability in bone mineral density.

However, certain limitations must be acknowledged. This is a cross-sectional analysis, which limits causal inference. Given the cross-sectional nature, temporal relationships cannot be established, and associations may reflect shared underlying frailty rather than direct effects. Additionally, the reliance on hospital-based data may introduce selection bias, as hospitalized patients may have inherently different osteoporosis risk profiles than the general elderly population. The study also did not account for dietary and lifestyle factors, which could influence BMD outcomes. Future longitudinal studies should explore whether improvement in functional and psychological domains translates into measurable gains in bone density or reduced fracture incidence.

## Conclusion

Our study demonstrates important associations between functional status, depressive symptoms and lower bone mineral density in hospitalized older adults. Because of the cross-sectional design, causality cannot be inferred. Routine assessments incorporating mental health and functional status may improve identification of patients at increased osteoporosis risk. These findings support integrating functional and psychological assessments into osteoporosis care pathways, a perspective aligned with the International Osteoporosis Foundation’s holistic approach to fracture prevention. Future research should focus on prospective interventions aimed at mitigating the effects of hospitalization and cognitive decline on bone health.

## Supplementary information

Below is the link to the electronic supplementary material.

Supplementary material 1

## Data Availability

No datasets were generated or analysed during the current study.
