# Supplementary material for: Functional status and depressive symptoms in relation to bone mineral density and osteoporosis in hospitalized older adults: a cross-sectional study
Source: BMC Geriatr. 2026 Oct 6;26:1260. doi: 10.1186/s12877-026-08424-4 (PMC13640116; doi:10.1186/s12877-026-08424-4)
Supplement: Supplementary file 1 [file 12877_2026_8424_MOESM1_ESM.docx]

**Supplementary material**

**Figure 1. Barthel Index and Fall Risk**. This bar chart compares the Barthel Index scores of individuals who experienced falls versus those who did not. Fallers exhibit significantly lower Barthel Index scores, indicating reduced functional status and greater dependence in daily activities. In contrast, non-fallers have higher scores, suggesting greater independence and mobility.
